# Biocompatible FePO_4_ Nanoparticles: Drug Delivery, RNA Stabilization, and Functional Activity

**DOI:** 10.1186/s11671-021-03626-8

**Published:** 2021-11-27

**Authors:** Sagar Rayamajhi, Sarah Wilson, Santosh Aryal, Robert DeLong

**Affiliations:** 1 Department of Chemistry, Kansas State University, Manhattan, KS 66502 USA; 2 Nanotechnology Innovation Center of Kansas State, College of Veterinary Medicine, Kansas State University, Manhattan, KS 66502 USA; 3 Department of Pharmaceutical Sciences and Health Outcomes, The Ben and Maytee Fisch College of Pharmacy, The University of Texas at Tyler, Tyler, TX 75799 USA

**Keywords:** Iron phosphate nanoparticles, Doxorubicin, Drug delivery, Drug loading, RNA stabilization, Biocompatible nanoparticles

## Abstract

FePO_4_ NPs are of special interest in food fortification and biomedical imaging because of their biocompatibility, high bioavailability, magnetic property, and superior sensory performance that do not cause adverse organoleptic effects. These characteristics are desirable in drug delivery as well. Here, we explored the FePO_4_ nanoparticles as a delivery vehicle for the anticancer drug, doxorubicin, with an optimum drug loading of 26.81% ± 1.0%. This loading further enforces the formation of Fe^3+^ doxorubicin complex resulting in the formation of FePO_4_-DOX nanoparticles. FePO_4_-DOX nanoparticles showed a good size homogeneity and concentration-dependent biocompatibility, with over 70% biocompatibility up to 80 µg/mL concentration. Importantly, cytotoxicity analysis showed that Fe^3+^ complexation with DOX in FePO_4_-DOX NPs enhanced the cytotoxicity by around 10 times than free DOX and improved the selectivity toward cancer cells. Furthermore, FePO_4_ NPs temperature-stabilize RNA and support mRNA translation activity showing promises for RNA stabilizing agents. The results show the biocompatibility of iron-based inorganic nanoparticles, their drug and RNA loading, stabilization, and delivery activity with potential ramifications for food fortification and drug/RNA delivery.

## Introduction

Among various inorganic nanoparticles such as gold, silica, and quantum dots, iron-based nanoparticles (Fe-NPs) are widely explored for biomedical applications like contrast agents, drug delivery vehicles, and thermal-based therapeutics [1–3]. Owing to the magnetic property, high bio-adaptability, and known endogenous metabolism of iron, Fe-NPs are desirable candidates for biomedical applications. As such, Fe-NPs make the majority of FDA-approved inorganic nanomedicine [1, 2]. These include INFeD, DexFerrum, Ferrlecit, Venofer, Feraheme, and Injectafer which are commercially available for their application in iron-deficient anemia and iron deficiency in chronic kidney disease [1]. Similarly, intravenous administration of the chelate iron gluconate is a well-tolerated intervention for anemia [4]. Anemia is one of the most prevalent nutritional deficiency in the world and Fe-based nanoparticles like FePO_4_ and FeSO_4_ has been used in food fortification to prevent anemia. Food fortification is the process of adding micronutrients to the food with an aim to overcome the nutritional deficiency in a population [5]. FePO_4_ NPs are of special interest in food fortification because of their biocompatibility, high bioavailability, and superior sensory performance that do not cause adverse organoleptic effects [6–9]. Perfecto et al. have demonstrated the FePO_4_ NPs internalization in human intestinal cells occurs primarily through divalent metal transporter-1 (DMT-1) and therefore can be readily absorbed [9, 10]. Iron-based Feridex® and Revosit® are widely used magnetic resonance imaging (MRI) contrast agents for contrast enhanced MRI [11–16]. In light of these outstanding reports, FePO_4_ NPs present themselves as a good delivery vehicle. Here, we explored FePO_4_ as a drug-delivery vehicle by loading an anticancer drug, Doxorubicin (DOX). Ferric ion (Fe^3+^) can form complex with DOX molecule facilitated by electrostatic interaction between electron deficient Fe in FePO_4_ and electron rich –OH group in DOX to form DOX loaded FePO_4_ NPs: FePO_4_-DOX NPs. We evaluated the physicochemical properties of FePO_4_ and FePO_4_-DOX NPs and assessed their biocompatibility and cytotoxicity profile, respectively, in mouse osteosarcoma K7M2 and fibroblast NIH/3T3 cell-line.

Along with that, the inorganic nanoparticle has shown promises in nucleic acid stabilization and delivery [17–19]. In this regard, the gold nanoparticle has been widely studied because of their ability to immobilize oligonucleotides in their surface resulting in the prevention of molecular aggregation and degradation [17, 20]. However, gold is not an endogenous element and thereby may limit its translational application. Here, Fe-based nanoparticles like FePO_4_ nanoparticles can be of prime interest for RNA stabilization study because of their endogenous nature and established biocompatibility profile. There is two proposed mechanism of interaction of nucleic acid (RNA/DNA) with Fe-NPs for the stabilization—(1) formation of hydrogen bonds and electrostatic interaction between the phosphate group of nucleic acid backbone and Fe-NPs resulting in adsorption of nucleic acid in Fe-NPs, and (2) nucleic acid can  adsorb to the Fe-NPs surface via nucleotide base pair interaction [19, 21, 22]. A study has shown the potential of calcium phosphate nanoparticles for DNA vaccine stabilization and delivery [23]. In this regard, here we have explored the RNA stabilization and functional activity of another phosphate-based nanoparticle, FePO_4_, to investigate the multifunctional potential of FePO_4_ based nanoparticles, in the delivery and stabilization of cargo.

With rapid approval of mRNA vaccine against COVID19, mRNA vaccine nanoparticles are of great interest, RNA being subject to rapid hydrolysis and loss of functional expression, it is incumbent upon the nanoparticle to improve these critical characteristics. Here we show FePO_4_ NPs stabilize RNA and support functional mRNA translation. Given these excellent characteristics, FePO_4_ NPs may merit consideration for food fortification, drug, and RNA delivery, opening up exciting biomedical applications.

## Results and Discussion

### FePO_4_ NPs Synthesis, Characterization, and Biocompatibility Analysis

**Fig. 1:**
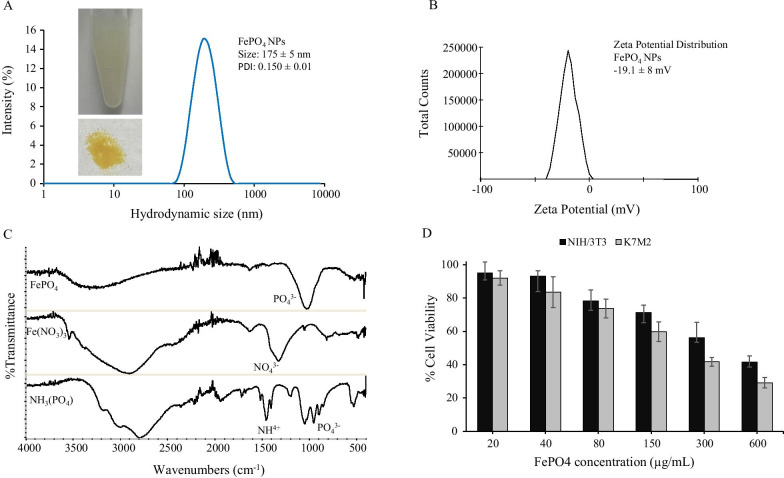
Characterization and biocompatibility of FePO_4_ nanoparticles. **a** Hydrodynamic size distribution of FePO_4_ NPs, **b** zeta potential measurement of FePO_4_ NPs showing the surface charge, **c** FTIR of FePO_4_ NPs and its precursor-Fe(NO_3_)_3_ and (NH_4_)_3_PO_4_, and **d** biocompatibility of FePO_4_ NPs on mouse osteosarcoma K7M2 and mouse fibroblast NIH/3T3 cell line. NPs were treated at various concentrations for 48 h (**a, b** data represents mean ± s.d.; *n* = 3 replicates. **d** represents mean ± s.d., *n* = 6 replicates).

A simple one-step chemical reaction between (NH_4_)_3_PO_4_ and Fe(NO_3_)_3_ gives FePO_4_ as precipitate which is dispersed in biocompatible lipid-PEG surfactant that helps to stabilize FePO_4_ nanoparticles and prevent aggregation. FePO_4_ NPs showed a hydrodynamic size of 175 ± 5 nm with a polydispersity index (PDI) of 0.150 ± 0.01 suggesting good particle homogeneity and narrow size distribution. Zeta potential analysis showed a negative surface charge of FePO_4_ NPs with − 19.1 ± 8 mV zeta potential. The negative surface charge further helps to stabilize particles in colloids thereby preventing protein opsonization, a mechanism that prevents cellular targeting and alters pharmacokinetics [24–26]. FePO_4_ was further characterized by FTIR. Figure 1c shows the spectral characteristic of FePO_4_ nanoparticles and their precursor—Fe(NO_3_)_3_ and (NH_4_)_3_PO_4_. FePO_4_ spectra show a distinct sharp peak on 1030 cm^−1^ which can be attributed to the P–O stretching band, a small peak at 520 cm^−1^ corresponds to the O–P–O antisymmetric bending, and a broad ranges from 3000 to3500 cm^−1^ represents water bending and stretching vibrations from adsorbed water molecules [27, 28]. The FePO_4_ spectra showed the presence of PO_4_^3−^ group and are similar to the FTIR peak reported by other studies thus confirming the formation of FePO_4_ nanoparticles [27–29]. Fe(NO_3_)_3_ spectra showed characteristic peaks for N–O stretching bands at 1326 and 813 cm^−1^ [30]. Peak at 1625 can be attributed to –OH bending vibration and a broad peak around 3000 cm^−1^ can be attributed to water bending and stretching vibrations [30]. Likewise, (NH_4_)_3_PO_4_ showed characteristic peaks for the ammonium group around 1500 cm^−1^ and phosphate group around 1000 cm^−1^ [31]. The absence of nitrate and ammonium peaks in FePO_4_ nanoparticles suggest the product is free from possible byproducts and confirms the purity of synthesis.

With the assurance of successful synthesis, purity, good size homogeneity, and stable surface charge of FePO_4_ NPs, we went on to analyze the biocompatibility of FePO_4_ NPs. For this purpose, we used cancer and non-cancer cells: mouse osteosarcoma K7M2 and mouse fibroblast NIH/3T3 and analyzed the biocompatibility of NPs at a varying concentration in terms of cell viability using MTT assay. FePO_4_ NPs showed concentration-dependent biocompatibility in both cell lines-K7M2 and NIH/3T3, in the concentration range of 20 to 600 µg/mL (Fig. 1d). FePO_4_ NPs showed good biocompatibility up to 80 µg/mL concentration with cell viability greater than 70%. Biocompatibility was relatively higher in non-cancer cell NIH/3T3 compared to cancer cell K7M2.

### Doxorubicin Loading in FePO_4_ and Cytotoxicity of FePO_4_-DOX

**Fig. 2:**
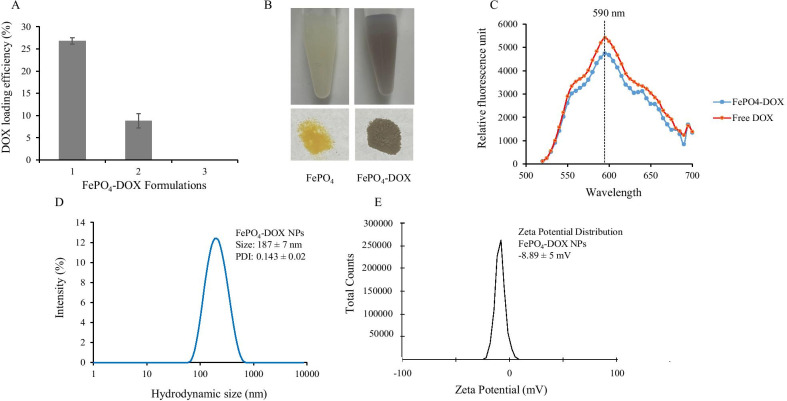
Doxorubicin (DOX) loading in FePO_4_ NPs and characterization of FePO_4_-DOX. **a** DOX loading efficiency in three different formulations of FePO_4_ NPs and DOX, **b** pictorial representation of the change of color from yellow to brown after DOX loading in FePO_4_ to formulate FePO_4_-DOX, **c** emission spectra characterization of DOX loaded FePO_4_ NPs (FePO_4_-DOX) after excitation at 480 nm, **d** hydrodynamic size distribution of FePO_4_-DOX NPs, and **e** zeta potential characterization of FePO_4_-DOX NPs showing surface charge (data represents mean ± s.d.; *n* = 3 replicates)

Doxorubicin is loaded in FePO_4_ through the co-incubation-precipitation method in which doxorubicin solution is mixed with the precursor of FePO_4_ that results in the formation of DOX loaded FePO_4_. Three different formulations to load DOX are employed as discussed in the methods. Formulation 1 showed the best loading efficiency of 26.81% ± 1 whereas formulation 2 showed a loading efficiency of 8.83% ± 2 and formulation 3 did not show any loading (Fig. 2a). For loading, we added DOX solution to the precursor Fe(NO_3_)_3_ in formulation 1 and to (NH_4_)_3_PO_4_ in formulation 2, whereas, in formulation 3, we added DOX solution to FePO_4_ NPs directly. The loading data clearly showed that adding DOX to the FePO_4_ NPs does not retain the DOX whereas adding DOX to either precursor: Fe(NO_3_)_3_ and (NH_4_)_3_PO_4_ solution helps in the loading and retention of DOX. This can be explained by the fact that Fe^3+^ from Fe(NO_3_)_3_ can form a complex with the electron-rich oxygen group present in Doxorubicin [32, 33]. The Fe^3+^-DOX complex is then precipitated by the addition of (NH_4_)_3_PO_4_ resulting in FePO_4_-DOX, which is characterized by a change of color from faint yellow to faint brown (Fig. 2b). Despite the color change, there was no change in the emission spectra of FePO_4_-DOX which showed emission maxima at 590 nm similar to that of Free DOX, when excited at 480 nm (Fig. 2c). FePO_4_-DOX NPs showed a hydrodynamic size of 187 ± 7 nm and PDI of 0.143 ± 0.02, similar to that of FePO_4_ (Fig. 2d). However, there was a significant difference in the surface charge of FePO_4_-DOX NPs (-8.89 ± 5 mV), compared to FePO_4_ NP (-19.1 ± 8 mV) (Fig. 2e). Change in zeta potential suggests functional changes in the surface property of nanoparticles. Here, the reduction of zeta potential from − 19.1 to − 8.89 mV can be attributed to the DOX complexation which adds cationic property in the complex.

**Fig. 3:**
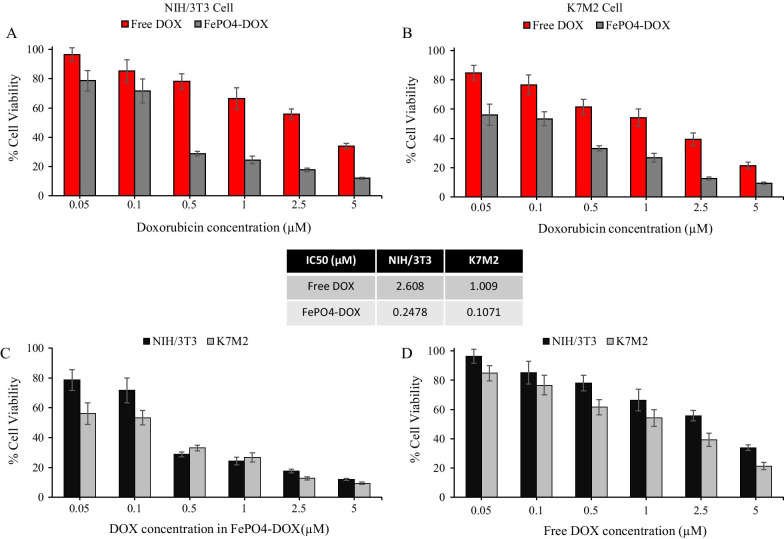
Cytotoxicity of FePO_4_-DOX NPs. **a, b** Cytotoxicity of Free Doxorubicin (DOX) and FePO_4_-DOX NPs in mouse fibroblast NIH/3T3 and osteosarcoma K7M2 cell-line, respectively, at various DOX equivalent concentration. Cytotoxicity was analyzed in percentage cell viability after particle treatment for 48 h. **c, d** A comparison in percentage cell viability of FePO_4_-DOX NPs and Free DOX in NIH/3T3 and K7M2 cell lines, respectively, at equivalent DOX concentration. The inset in the middle represents the IC-50 values of Free DOX and FePO_4_-DOX NPs in NIH/3T3 and K7M2 cells (data represents mean ± s.d.; *n* = 6 replicates)

Following the physicochemical characterization, the cytotoxicity of FePO_4_-DOX was analyzed in K7M2 and NIH/3T3 cells and compared with free DOX (Fig. 3). FePO_4_-DOX showed higher cytotoxicity compared to Free DOX at equivalent DOX concentration in both cell lines. IC50 value showed around 10 times reduction with FePO_4_-DOX treatment, from 2.61 to 0.248 µM in NIH/3T3 and 1.01 to 0.107 µM in K7M2 cells. This drastic reduction in IC50 value in both cell lines suggests an enhanced cytotoxicity profile of FePO_4_-DOX NPs. The equivalent FePO_4_ concentration in the IC50 concentration range of FePO_4_-DOX is 40 µg/mL (0.107 µM in K7M2 cells) and 100 µg/mL (0.248 µM in NIH/3T3 cells), which are both within the biocompatible range of FePO_4_ concentration, with more than 70% cell viability. Hence, the elevation of FePO_4_-DOX cytotoxicity can be attributed to the Fe^3+^-DOX complex formation and not to the individual contribution of FePO_4_ and DOX. Literature has shown the elevated cytotoxic effect of anthracycline like doxorubicin in presence of iron [34–37]. These reports are further supported by the alleviation of Fe-DOX cytotoxicity by the use of iron chelators [35–37]. One proposed mechanism is Fe-DOX complex potentiates the toxicity of DOX-derived reactive oxygen species (ROS) transforming relatively safe ROS (O^2·^– and H_2_O_2_) into much more toxic ROS leading to elevated DNA damage and cell death [34, 36]. Another proposed mechanism is the interaction of DOX with the function of iron regulatory proteins and ferritin in presence of excess Fe thereby affecting iron homeostasis leading to ROS-dependent and independent damage and apoptotic cell death [36, 38].

Along with the elevated cytotoxicity, FePO_4_-DOX showed selectivity toward cancer cells with higher cytotoxicity behavior similar to that of Free DOX. Figure 3c shows 0.1 µM DOX equivalent FePO_4_-DOX showed 53% cell viability for cancer cell K7M2 compared to 72% cell viability for non-cancer NIH/3T3. Likewise, Free DOX also showed higher cytotoxicity behavior toward cancer cells, with 54% cell viability in K7M2 cells compared to 66% in NIH/3T3. However, the differences have increased in the case of FePO_4_-DOX, with 19% differences in cell viability among cancer and non-cancer cells compared to 12% in Free DOX. Cytotoxicity analysis has shown that Fe complexation with DOX in FePO_4_-DOX NPs has significantly enhanced the cytotoxicity and improved the selectivity toward cancer cells.

### Cellular Internalization of FePO_4_-DOX NPs

**Fig. 4:**
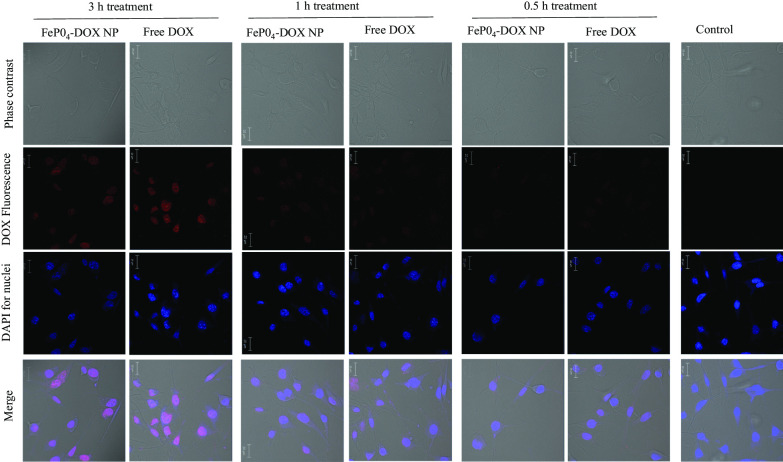
Cellular internalization study. Cellular internalization of FePO_4_-DOX NPs and Free DOX on K7M2 cells after 3 h, 1 h, and 0.5 h treatment. Cells were treated with 200 µL of 5 µg/mL DOX concentration. The red color observed in nanoparticle treated cell line signify successful internalization of the nanoparticles. The red color is due to the fluorescence characteristic of DOX. No red signal is observed in the untreated control cell

The internalization behavior of FePO_4_-DOX NPs was analyzed using confocal microscopy following a time-dependent internalization study (Fig. 4). Free DOX was used as a positive control. Both FePO_4_-DOX NPs and Free DOX did not show significant internalization in the initial 0.5 and 1 h incubation time points. However, at 3 h incubation, both showed internalization as depicted by red DOX fluorescence in the confocal image. The blue color comes from nucleus staining by DAPI. The analysis shows that within 3 h, FePO_4_-DOX NPs internalize to cells following similar internalization behavior as that of Free DOX. It is important to note that, due to the change of color of FePO_4_-DOX, which is brownish compared to the red color of Free DOX, we may not quantitatively compare the relative internalization profile of FePO_4_-DOX. Nonetheless, the internalization assay confirmed that FePO_4_-DOX is uptake by the cells within 3 h. Given the well-understood mechanism of handling iron by our body, proposed NPs could hold promises in the development of iron-based anticancer therapeutics with an ability to monitor therapeutic response in a single therapy session.

### RNA Stabilization and mRNA Expression

**Fig. 5:**
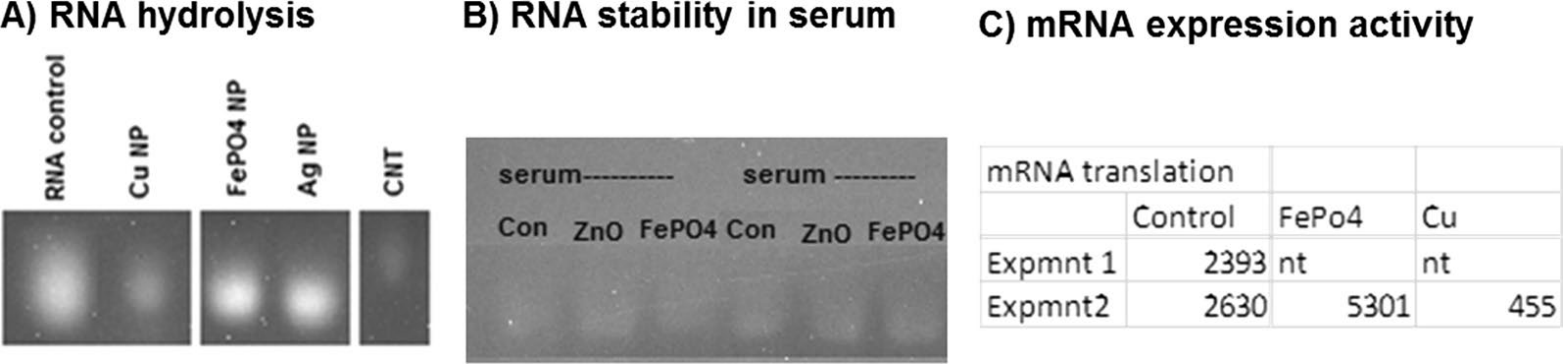
RNA hydrolysis. **a** A model RNA we have used in a number of our publications similar in size and sequence composition to most mRNAs from torula yeast (TY-RNA) was used. The RNA was incubated in double-distilled water over time in the presence or absence of nanoparticles either copper (Cu NP), iron phosphate (FePO4), silver (Ag NP) or carbon nanotube (CNT) at 37 degrees celcius and samples removed at the same time point and assayed by RNA agarose gel electrophoresis (RAGE). Loss of band staining intensity indicates RNA degradation whereas maintenance of RNA band staining intensity indicates stabilization. **b** Similar to above, RNA was incubated in 10% FBS/DMEM at room temperature in the presence of zinc oxide (ZnO) NP or FePO_4_ NP versus control which was RNA alone in the absence of nanoparticle. Again samples were removed over time and assayed by RAGE, presence of the stained RNA band over time again indicates stability and resistance from nuclease or RNase degradation from the serum. **c** mRNA encoding Luciferase was translated in vitro from standard rabbit reticulocyte and the relative luminescence standardized to RNA in the presence or absence of either iron phosphate (FePO_4_) or copper (Cu) nanoparticle

As can be seen in Fig. 5a, whereas copper nanoparticles (Cu NP) and carbon nanotubes (CNTs) accelerate RNA hydrolytic degradation (lower band intensity than control), the FePO_4_ and control silver (Ag) nanoparticle stabilize the RNA as shown by relatively strong band intensity in RNA agarose gel electrophoresis (RAGE). The FePO_4_ and control zinc oxide nanoparticle (ZnO NP) also impart some resistance to degradation in serum, as depicted by the band intensity which is slightly higher than controls (Fig. 5b). Importantly the functional activity, mRNA expression is higher than non-nanoparticle controls, whereas the RNA-degrading Cu NP causes loss in mRNA expression as measure by relative light units (Fig. 5c). These results show that FePO_4_ NPs helps to stabilize RNA and can be used as a stabilizing delivery agent for therapeutic RNA delivery. Earlier preliminary experiments had indicated a normal working range of translation shown are two independent experiments for the control non-nanoparticle treated samples showing 2393 and 2630 RLU/well which is representative. A twofold increase consistent with the above data suggests FePO44 NP supports translation whereas consistent with RNA denaturation/degradation above, the Cu NP suppresses translation. A variety of inorganic nanoparticle systems have been exploited for therapeutic RNA stabilization and delivery including; gold, silver, copper, iron oxide, mesoporous silica nanoparticle (MSN), carbon-based polymers, composites, and others [39–45]. For example our group had reported nanoparticle complexation to macromolecular RNA can cause it to resist degradation by RNase, or nucleases present in serum and tissues. The COVID-19 mRNA vaccine has renewed interest in such macromolecular RNA therapies extending beyond vaccines, where it is incumbent upon the nanoparticle to not only protect RNA from hydrolysis and nuclease-mediated digestion, but complexation to the NP must preserve RNA function, for example, mRNA expression. Previously we had seen copper nanoparticle complexation macromolecular RNA causes RNA denaturation [46] Thus we investigated the effects of NP complexation to macromolecular RNA using Torula yeast RNA (TY-RNA) or a reporter construct mRNA expressing Luciferase.

## Conclusion

FePO_4_ nanoparticles were successfully synthesized following a simple co-incubation-precipitation technique resulting in the formation of homogenous-sized particles of 175 ± 5 nm. FTIR analysis confirmed the presence of phosphate group and the absence of precursor impurities in the nanoparticle. Biocompatibility analysis revealed concentration-dependent biocompatibility with more than 70% cell viability up to 80 µg/mL. Further, DOX was effectively loaded in FePO_4_ resulting in FePO_4_-DOX NPs which showed similar physicochemical properties to that of FePO_4_. Cytotoxicity analysis revealed that Fe complexation with DOX in FePO_4_-DOX NPs enhanced the cytotoxicity, with around 10 times improvement in IC50, and improved the selectivity toward cancer cells. Additionally, the internalization assay showed FePO_4_-DOX NPs were efficiently internalized in cells at a 3 h incubation time point. RNA stabilization study showed that FePO_4_ nanoparticles efficiently stabilize RNA, prevent rapid degradation, and maintain the functional activity demonstrating promises for delivery of therapeutic RNA. Given the good size homogeneity, biocompatibility range, drug loading efficiency, enhanced cytotoxicity profile, RNA stabilizing property, and efficient cellular uptake, FePO_4_ NPs showed desirable characteristics for drug and RNA delivery vehicles. Furthermore, the results have shown promising prospects of using FePO_4_-drug NPs in food fortifications for the development of a food-based drug platform.

## Methods

### Synthesis and Characterization of FePO_4_ Nanoparticles

FePO_4_ nanoparticles were synthesized by chemical precipitation technique optimizing protocol by Sokolova et al. [47]. Briefly, Ammonium phosphate ((NH_4_)_3_PO_4,_ 16 mg/mL) and iron nitrate (Fe(NO_3_)_3,_ 8 mg/mL) solution was prepared. To the 1 mL of Fe(NO_3_)_3_, 1 mL of (NH_4_)_3_PO_4_ was added dropwise under constant stirring resulting in precipitation of iron phosphates (FePO_4_). Excess of (NH_4_)_3_PO_4_ was used so that all Fe from Fe(NO_3_)_3_ precipitate as FePO_4._ Thus formed iron phosphates solution was washed with water 3 times to remove byproducts by centrifuging at 300 g for 2 min. Finally, FePO_4_ precipitate was dispersed with DSPE-PEG-COOH solution (10% w/w) in water to formulate FePO_4_ nanoparticles. FePO_4_ NPs were characterized for size and surface property using dynamic light scattering (DLS) and spectral characteristics using Fourier Transform Infrared Spectroscopy (FTIR).

### Doxorubicin (DOX) Loading on FePO_4_ Nanoparticles

Doxorubicin was loaded in FePO_4_ nanoparticles by the co-incubation-precipitation method. Three different DOX-FePO_4_ NPs formulations were explored to optimize the best loading efficiency. In the first formulation, DOX-FePO_4_ NPs were formulated by adding 100 µg DOX in 1 mL of Fe(NO_3_)_3_ (8 mg/mL) followed by the addition of 1 mL of (NH_4_)_3_PO_4_ (16 mg/mL) dropwise under constant stirring. In the second formulations, 100 µg DOX was first added to 1 mL of (NH_4_)_3_PO_4_ (16 mg/mL) followed by the addition of 1 mL of Fe(NO_3_)_3_ (8 mg/mL) dropwise under constant stirring. In the third formulations, 100 µg DOX was added to the FePO_4_ NP solution. Thus formulated FePO_4_-DOX NPs were washed three times with water and the amount of doxorubicin in FePO_4_-DOX was quantified spectrofluorimetrically by measuring DOX excitation and emission at 490 nm and 595 nm.

DOX loading efficiency was calculated by the following equation:

$$\% \;{\text{Loading}}\;{\text{efficiency:}}\;\left( {{\text{DOX}}\;{\text{ present}}\;{\text{ in}}\;{\text{ FePO}}_{{4}} - {\text{DOX}}\;{\text{NP/Initial}}\;{\text{input}}\;{\text{of}}\;{\text{DOX}}} \right) \times {1}00$$

### Biocompatibility of FePO_4_ NPs and Cytotoxicity of FePO_4_-DOX NPs

Biocompatibility of FePO_4_ NPs and cytotoxicity of FePO_4_-DOX NPs were assayed in mouse osteosarcoma K7M2 and mouse fibroblast NIH/3T3 using MTT assay following established protocol [48, 49]. Briefly, 10,000 cells were seeded in 96 well plates and incubated for 24 h in a 37 °C 5% CO_2_ incubator. Then, media was removed and fresh media with varying concentrations of nanoparticles were treated to cells and left for incubation for 48 h. Control cells were maintained with media only. FePO_4_ NPs concentration ranges from 20 to 600 µg/mL and DOX concentration ranges from 0.05 to 5 µM. After NP incubation, media was removed and cells were incubated with MTT solution (0.5 mg/ml) in serum-free media for 2 h to allow for the formation of formazan crystal. MTT solution was removed and formazan crystal was dissolved in DMSO and left for 15 min at room temperature for proper mixing. Then the absorbance of DMSO solution was measured at 550 nm using a microplate reader (BioTek, Synergy H1 Hybrid Reader) and percentage cell viability was calculated.

### Cellular Internalization via Confocal Microscopy

Cellular internalization of FePO_4_-DOX NPs was analyzed in mouse osteosarcoma K7M2 cells using confocal microscopy [49–51]. Briefly, 12,000 cells were seeded in 8-well plates and incubated for 24 h in 37 °C 5% CO_2_ incubator. Then, 200 µL of 5 µg/mL DOX concentration in media were treated for 3 h, and cells were fixed with 4% Paraformaldehyde for imaging. The nucleus was stained by DAPI and cells were observed under a Confocal Laser Scanning Microscope (Carl Zeiss, LSM-700). Here, the emission maximum of DOX at 560 nm can be exploited to track its internalization which gives red color in confocal microscopy. Using the same protocol, a time-dependent internalization assay was performed by incubating FePO_4_-DOX NPs and Free DOX for 0.5, 1, and 3 h respectively.

### RNA Stability and Expression

Torula yeast RNA (Sigma-Aldrich) was dissolved at 1 mg/ml in sterile deionized water and 2 µg aliquots exposed to 20 ug/mL nanoparticle (CNT, Cu, Ag, ZnO NP or FePO_4_) incubated at 37 deg C and assayed over time by RNA agarose gel electrophoresis as we have previously reported [42, 52]. Timepoint shown in Fig. 5 is overnight. Similarly, the RNA with/without nanoparticles was exposed to 10% FBS/DMEM and again assayed by RAGE as above. mRNA fLuc was obtained from Trilink Biotechnologies, 2 µl were incubated in rabbit reticuloysate supplemented with Methinine, Cysteine and Leucine (ProMega Corp) for 30 degrees for 1.5 h with or without nanoparticle at 20 μg/ml, standard Luciferin reagent added, and luminescence measurement taken on a Biotek Synergy H1 plate reader under standard conditions.

### Statistical Analysis

All data represents at least three independent replicates and expressed as mean ± s.d. whenever possible. Cell viability data includes six replicates.

## Data Availability

The datasets used and/or analyzed during the current study are available from the corresponding author on reasonable request.
